# Cost-effectiveness analysis of pembrolizumab versus chemotherapy in advanced non-small cell lung cancer in China based on real-world studies

**DOI:** 10.1007/s00432-025-06242-6

**Published:** 2025-10-28

**Authors:** Ning Wan, Chen Yang, Bing Wang, Ya Guo, ZiJian He, YaJuan Lv, LiQing Lu, Ning Yang, WeiBin Xiao, YongBang Chen, Jin Yuan, DanDan Yang, Tao Liu, WenFeng Fang, ZhuoJia Chen, WeiTing Liang

**Affiliations:** 1 Department of Clinical Pharmacy, General Hospital of Southern Theater Command, Guangzhou, 510010 China; 2 Department of Pharmacy, Guangzhou University of Chinese Medicine, Guangzhou, 510006 China; 3 School of Pharmaceutical Sciences, Southern Medical University, Guangzhou, 510515 China; 4 Department of Pharmacy, Heyou Hospital, Shunde District, Foshan, 528306 China; 5 College of Pharmacy, Jinan University, Guangzhou, 510623 China; 6 Department of Oncology, General Hospital of Southern Theater Command, Guangzhou, 510010 China; 7 Department of Pharmacy, The First Affiliated Hospital of Guangzhou Medical University, Guangzhou, 510120 China; 8 School of Pharmaceutical Science, Sun Yat-sen University, Guangzhou, 510006 China; 9 Department of Pharmacy, State Key Laboratory of Oncology in South China, Guangdong Provincial Clinical Research Center for Cancer, Sun Yat-sen University Cancer Center, 651 Dongfeng Road East, Guangzhou, 510060 China; 10 Department of Medical Oncology, State Key Laboratory of Oncology in South China, Collaborative Innovation Center for Cancer Medicine, Sun Yat-sen University Cancer Center, 651 Dongfeng Road East, Guangzhou, 510060 China

**Keywords:** Real-world studies, Pembrolizumab, Non-small cell lung cancer, Partition survival model, Cost-effectiveness analysis

## Abstract

**Background:**

Although pembrolizumab has been shown to be effective, its high price has prevented it from being widely used. Especially in the real world, the application situation is still uncertain. The purpose of this study was to evaluate the cost-effectiveness of pembrolizumab on the basis of real-world studies, from the perspective of the health care system.

**Methods:**

Retrospectively, 630 patients with advanced NSCLC treated with pembrolizumab (monotherapy or combination chemotherapy) versus chemotherapy alone from January 2020 to December 2022 at a large 3 A hospital in China were included. Confounders between groups were eliminated using propensity score matching analysis. A partitioned survival model was developed to evaluate the cost-effectiveness of pembrolizumab versus chemotherapy for the treatment of advanced NSCLC based on progression-free survival, overall survival, and the incidence of adverse effects in the two matched groups (*n* = 450 patients). The incremental cost-effectiveness ratio was calculated. The impact of a drug donation program on the cost-effectiveness of pembrolizumab was also evaluated.

**Results:**

Pembrolizumab significantly improved median PFS in patients (15.5 months vs. 8.8 months). The median OS in the Pembrolizumab group has not been reached, while it was 26.2 months in the chemotherapy group. When the drug donation program is not considered, the ICER of pembrolizumab is $146,409.07/QALY. Regardless of whether the willingness-to-pay threshold is set at three times the per capita GDP of China ($36,070.2) or three times the per capita GDP of Guangdong Province ($64,523.8), the use of pembrolizumab is not cost-effective. However, after considering the drug donation program, the ICER decreased to $56,127.74/QALY. Under the willingness-to-pay threshold of three times the per capita GDP of Guangzhou in 2022 ($64,523.8), pembrolizumab became a cost-effective choice.

**Conclusion:**

In the treatment of advanced NSCLC in China, pembrolizumab, particularly when considering the drug donation program, offers better survival outcomes and becomes cost-effective. This highlights the importance of such programs in making high-cost treatments accessible in real-world clinical settings.

**Supplementary Information:**

The online version contains supplementary material available at 10.1007/s00432-025-06242-6.

## Interoduction

Lung cancer remains the leading cause of cancer deaths worldwide, with non-small cell lung cancer (NSCLC) accounting for approximately 85% of all lung cancer types (Bray et al. 2024; Schenk et al. 2021) Conventional platinum containing chemotherapy is the standard first-line treatment for advanced NSCLC, however, its survival rate is relatively low (Gettinger et al. 2018; Rossi and Di Maio 2016). In the past few years, significant advancements in lung cancer treatment have been achieved through the development of immune checkpoint inhibitors (ICIs), specifically targeting programmed death receptor 1 (PD-1) and its ligand PD-L1. These ICIs work by impeding the interaction between PD-1 and PD-L1, effectively hindering the proliferation of cancer cells. Pembrolizumab, one of the ICI drugs, is proven to be clinically effective for advanced NSCLC in multiple randomized controlled trials (RCTs) and has become one of the clinical treatment options.

Despite the exciting results of this new immunotherapy. However, the high prices associated with these new therapies pose a significant challenge to the healthcare system. Relevant economic studies have shown that the current price tag of pembrolizumab is not cost-effective and that it needs to be reduced to be cost-effective compared to commonly used chemotherapy treatments (Criss et al. 2020; Hu and Hay 2018; Zeng et al. 2019; Zhou et al. 2019). However, all of the above pharmacoeconomic studies were based on the results of RCTs, and there is a noticeable scarcity of economic analyses based on outcomes from real-world studies.

While RCTs have shown the clinical benefits of using pembrolizumab alone or alongside chemotherapy in the management of advanced NSCLC, there may be some differences between the patients enrolled in RCTs and those in real-world situations due to its strict criteria. To date, there have been several real-world studies exploring the efficacy and safety of pembrolizumab, but few have been reported economically.

Therefore, it is also important to understand economics in real-world clinical settings. Good observational studies can also provide some degree of evidence to complement clinical trials. This study aimed to assess the cost-effectiveness of pembrolizumab, both as a single-agent therapy and in combination with chemotherapy, compared to chemotherapy alone in treating advanced NSCLC in real-world settings.

## Materials and methods

### General data

A retrospective study was conducted, collecting data from 630 patients with advanced non-small cell lung cancer (NSCLC) treated at Sun Yat-sen University Cancer Center from April 2017 to December 2022, and patients should undergo treatment within January 2020 to December 2022. Among them, 169 patients received pembrolizumab (monotherapy or combined with chemotherapy), and 461 patients received chemotherapy. The study was approved by the Ethics Committee of Sun Yat-sen University Cancer Center, with the ethics approval number B2022-153-01.

### Inclusion criteria

The inclusion criteria were: (1) 18 years or older, (2) Participants required a first-time diagnosis of stage III/IV NSCLC by imaging and pathological tissue, (3) At least one measurable lung lesion, (4) At least 2 weeks of treatment with pembrolizumab or conventional chemotherapy, (5) Routine baseline examinations must be performed during or before treatment with pembrolizumab or conventional chemotherapeutic agents: chest and abdomen scan + enhanced computed tomography (CT), complete biochemistry, routine blood work, cardiac enzymes, plasma adrenocorticotropic hormone, serum cortisol, two pancreatic enzymes, and so on, (6) Patients receiving pembrolizumab monotherapy or in combination with chemotherapy and platinum-based chemotherapy. The exclusion criteria were (1) Individuals diagnosed with additional malignant tumors, (2) Participants who had an expected survival of less than 1 month, (3) Patient adherence to treatment with poor compliance and incomplete medical records, (4) Patients in the pembrolizumab group received multiple ICIs, such as the use of nivolumab, atezolizumab, and so on.

### Efficacy and safety evaluation

Tumor response was evaluated based on the RECIST 1.1 criteria (Schwartz et al. 2016), including complete response (CR), partial response (PR), stable disease (SD), and progressive disease (PD). Progression-free survival (PFS) and overall survival (OS) for the three treatment groups were statistically analyzed. Adverse reactions and immune-related adverse reactions were evaluated and graded according to the National Cancer Institute Common Terminology Criteria for Adverse Events (CTCAE) Version 5.0 and the 2023 CSCO Guidelines for the Management of Toxicity Related to Immune Checkpoint Inhibitors.

### Confounding factors

General data such as gender and age between the two patient groups were compared. Prognostic factors affecting efficacy were selected as confounding factors. Propensity score matching was used to process the original data of the two groups to eliminate confounding factors between the groups. In this study, 1:3 nearest neighbor matching was done based on the PEM group, and the caliper value was set at 0.02.

### Treatment regimens

The treatment regimens used for patients in the two treatment groups after propensity score matching and subsequent treatment regimens after PD were organized to determine the weight of each regimen in the treatment group.

### Model construction

Based on the efficacy results of the two treatment groups after propensity score matching, a partitioned survival model was constructed using TreeAge Pro 2022 software. The model includes three mutually exclusive health states: PFS, PD, and death. The study model predicts over the patient’s lifetime, with treatment administered once every 3 weeks and discounted at a rate of 5%. Quality-adjusted life years (QALYs) were used as the health outcome indicator, comparing the incremental cost-effectiveness ratio (ICER) of the two treatment groups, with a willingness-to-pay (WTP) threshold set at three times the 2022 gross domestic product (GDP) of $36,070.2.

### Clinical data

The efficacy indicators for the economic evaluation of the two groups were derived from real-world studies. Individual survival data reconstruction and long-term survival assessment were performed on the K-M survival curves of the two groups after propensity score matching using R software 4.2.2 and the survHE package. The individual survival data were fitted to Exponential, Weibull, Log-logistic, and Logistic distributions. The goodness of fit was tested using Akaike Information Criterion (AIC) and Bayesian Information Criterion (BIC) to determine the reliability of the reconstructed K-M curves.

### Cost and utility data

 The cost data used in this study included direct medical costs: treatment drug costs, examination costs, supportive treatment costs, routine follow-up costs, subsequent drug treatment costs after disease progression, and expenses related to drug adverse events. Drug costs were obtained from the Guangdong GPO platform’s published prices. Costs for supportive treatment, adverse drug events, and related examinations were taken from published literature. Treatment regimens from the start of treatment to the first disease progression and subsequent treatment regimens after disease progression were derived from real-world data. Patient treatment regimens were organized and weighted to determine the contribution of each regimen to the overall treatment regimens in each group. For patients with disease progression whose subsequent treatment regimens were unknown, the costs were assumed to be supportive treatment costs. For patients who died during disease progression, the subsequent regimen costs were assumed to be $0. Additionally, the study included costs related to grade 3–5 adverse events in both groups, weighted by observed incidence. To simplify and reduce result bias due to chemotherapy drug dosage, a simulated patient with a body surface area of 1.80 m², weight of 65 kg, and creatinine clearance rate of 90 ml/min/1.73 m² was used to standardize chemotherapy drug doses (Zhu et al. 2021). Utility values for disease states and disutility values for adverse reactions were referenced from a utility value study of Chinese NSCLC patients conducted by Nafees et al (Nafees et al. 2017).

### Sensitivity analysis

The robustness of the model was determined through one-way sensitivity analysis and probabilistic sensitivity analysis. One-way sensitivity analysis tested the impact of each model parameter on ICER by varying it by ± 20% of its limit value, with results presented in a tornado diagram. Probabilistic sensitivity analysis used 1000 Monte Carlo simulations (cost data using Gamma distribution, utility values, and adverse event incidences using Beta distribution) to draw cost-effectiveness acceptability curves and cost-effectiveness scatter plots.

## Results

3.1| Characteristics of the patients 630 patients with advanced NSCLC were identified through an electronic database as having received pembrolizumab (monotherapy or combination chemotherapy) and chemotherapy. Among these individuals, 169 patients received pembrolizumab treatment, either alone or in combination with chemotherapy, while 461 patients underwent chemotherapy. The baseline demographic, clinical, and pathologic characteristics of the 630 patients were displayed in Table 1. The gender differences, TNM stage, pathological histology, the number of treatment lines (first/second-line treatment), and smoking status between the two groups before PSM were statistically significant (*p* < 0.05). After matching, the differences in other confounding variables between the two groups became statistically insignificant (*p* > 0.05).

**Table 1:** Baseline characteristics and treatment details of NSCLC patients receiving pembrolizumab or chemotherapy

Variables	Before PSM (*n* = 630)			After PSM (*n* = 450)		
	Patients treated with pembrolizumab group (*n* = 169)	Patients treated with chemotherapy group (*n* = 461)	*p*-value	Patients treated with pembrolizumab group (*n* = 149)	Patients treated with chemotherapy group (*n* = 301)	*p*-value
Age, years- Median (Interquartile)	61.0 (53.0–67.0)	60.0 (53.0–66.0)	0.325	60.0 (52.0–67.0)	61.0 (55.0-66.5)	0.492
BMI, Median (Interquartile)	22.8 (20.6–25.2)	23.1 (20.8–25.1)	0.703	22.8 (21.2–25.2)	22.9 (20.8–25.1)	0.345
Sex, n (%)			< 0.050			1.000
Male	134 (79.3%)	316 (68.5%)		117 (78.5%)	228 (75.7%)	
Female	35 (20.7%)	145 (31.5%)		32 (21.5%)	73 (24.3%)	
Histology subtype, n (%)			< 0.050			0.625
Squamous	52 (30.8%)	60 (13.0%)		39 (26.2%)	53 (17.6%)	
Non-Squamous	116 (68.6%)	396 (85.9%)		110 (73.8%)	248 (82.4%)	
NA	1 (0.6%)	5 (1.1%)				
TNM stage, n (%)			< 0.050			0.319
IV	125 (74.0%)	330 (71.6%)		112 (75.2%)	250 (83.1%)	
III	29 (17.1%)	109 (23.6%)		24 (16.1%)	35 (11.6%)	
NA	15 (8.9%)	22 (4.8%)		13 (8.7%)	16 (5.3%)	
ECOG PS at diagnosis, n (%)			0.817			0.455
0–1	141 (83.4%)	394 (85.5%)		125 (83.9%)	257 (85.4%)	
≥ 2	6 (3.6%)	14 (3.0%)		6 (4.0%)	7 (2.3%)	
NA	22 (13.0%)	53 (11.5%)		18 (12.1%)	37 (12.3%)	
Smoking status (%)			< 0.050			0.115
Previous Smoking	89 (52.7%)	186 (40.3%)		80 (53.7%)	153 (50.8%)	
Never smoked	62 (36.7%)	252 (54.7%)		61 (40.9%)	129 (42.9%)	
NA	18 (10.6%)	23 (5.0%)		8 (5.4%)	19 (6.3%)	
The number of treatment lines (%)			< 0.050			0.096
1	112 (66.3%)	344 (74.6%)		96 (64.4%)	203 (67.4%)	
≥ 2	57 (33.7%)	117 (25.4%)		53 (35.6%)	98 (32.6%)	

### Survival analysis

**Fig. 1:**
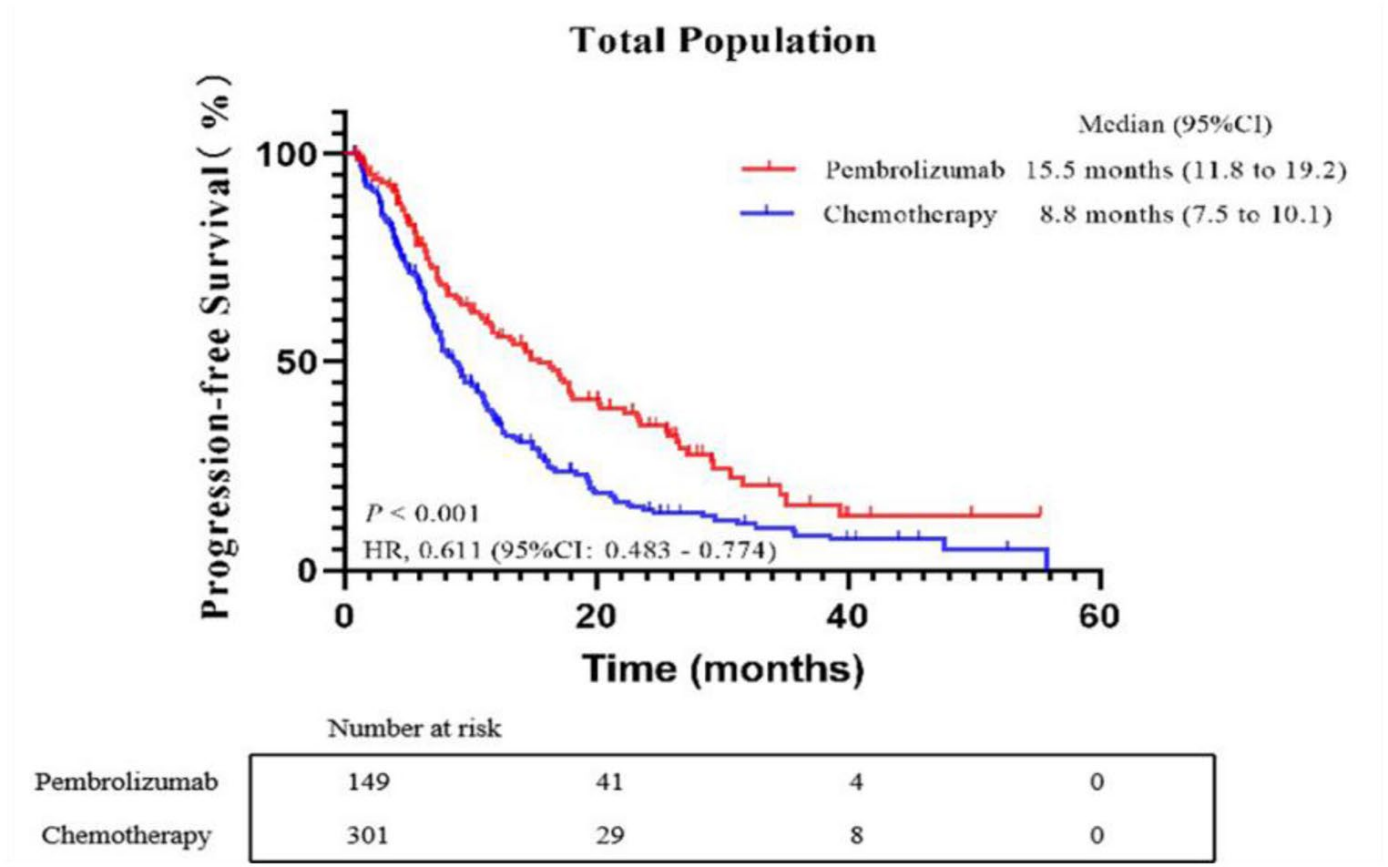
Kaplan-Meier curves of progression-free survival (PFS) comparing the pembrolizumab group and chemotherapy group. CI, confidence interval; HR, hazard ratio

**Fig. 2:**
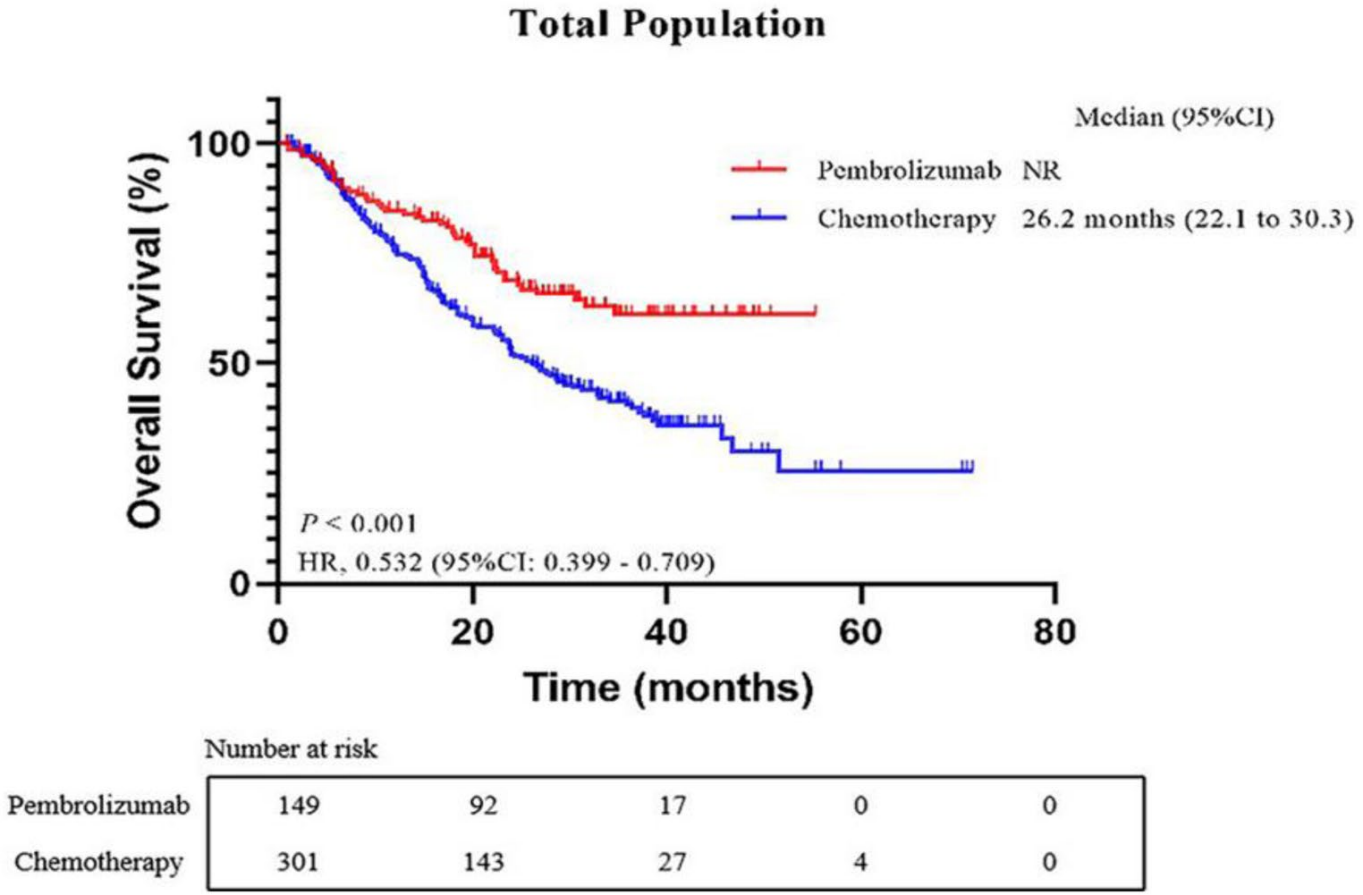
Kaplan-Meier curves of overall survival (OS) comparing the pembrolizumab group and chemotherapy group. CI, confidence interval; HR, hazard ratio

At the final evaluation point, out of the total patients, 281 (63.00%) experienced disease progression. Specifically, in the pembrolizumab group, 91 patients (62.33%) had disease progression, while in the chemotherapy group, this number was 190 (63.30%). The median PFS was notably different between the two groups: patients in the pembrolizumab group had a median PFS of 15.5 months (95%CI: 11.819.2), in contrast to the chemotherapy group, which had a median PFS of 8.8 months (95%CI: 7.510.1). This difference was statistically significant, with a Hazard Ratio (HR) of 0.611 (95% CI, 0.4830.774; *p* < 0.001), as illustrated in Fig. 1. For patients in the pembrolizumab group, the median OS had not been reached at the time of analysis. In contrast, for those in the chemotherapy group, the median OS was recorded at 26.2 months (95%CI: 0.483–0.774). The difference in survival outcomes between the two groups was significant, with an HR of 0.532 (95%CI, 0.399–0.709; *p* < 0.001), as depicted in Fig. 2.

### Cost-effectiveness analysis

The K-M survival plots for PFS and OS in both the pembrolizumab and chemotherapy groups were derived from the dataset. These plots were tailored to individual patient data, and the corresponding fitted outcomes can be found in Table 2. The best-fit distribution of the survival curves was judged based on the AIC value of each fitted distribution and the smaller the AIC value, the better the fit of the distribution. The best-fit distributions for the K-M survival curves of PFS and OS in both the pembrolizumab and chemotherapy groups were identified as log-normal distributions. The results of the fitted distribution parameters for each survival curve model are shown in Table 3.

The study encompassing 450 patients, which assessed the efficacy and safety of pembrolizumab and chemotherapy groups, compiled treatment regimens from the initiation of therapy to the first occurrence of PD. It also documented subsequent treatment strategies post-PD. The varieties of anticancer drugs utilized in these regimens are detailed in Additional file 1: Table S1. The cost of the relevant drugs was obtained from the Guangdong Provincial GPO platform. The expense for each cycle of various anticancer medications was determined using the treatment dosage guidelines for different drugs specified in the 2022 CSCO NSCLC treatment guidelines. This calculation was combined with the characteristics of a hypothetical patient, who has a Body Surface Area (BSA) of 1.80 m², weighs 65 kg, and has a Creatinine clearance rate (Ccr) of 90 ml/min/1.73 m². The upper and lower bounds of the drug costs were estimated by adjusting the drug cost by ± 20%. A uniform Gamma distribution was selected for the cost variability of drug treatments.

Drawing from the findings of the aforementioned safety assessment, significant non-immune-related adverse reactions observed in both the pembrolizumab and chemotherapy groups include anemia, neutropenia, and thrombocytopenia. These will be factored into the cost calculation for adverse reactions, incorporating negative utility values for such reactions. The costs for prevention and treatment of adverse reactions, screening, and supportive care described above were obtained from published literature and were shown in Additional file 1: Table S2.

The study calculated utility values for patients without disease progression and those with disease progression, as well as negative utility values for three severe adverse reactions: anemia, neutropenia, and thrombocytopenia. Both the utility values for patients and the negative utility values for adverse reactions were sourced from literature (Additional file 1: Table S3). All utility value data were subjected to ± 20% as upper and lower limits of utility values, and Beta distribution was chosen.

 Table  4 displays the outcomes of the cost-effectiveness analysis comparing the pembrolizumab group to the chemotherapy group, excluding the impact of any complimentary drug policy. For the chemotherapy group, the average cost per patient was $81,784, with an average QALY of 2.63. In contrast, the pembrolizumab group experienced an enhanced clinical benefit of 1.69 QALY, but at an increased cost of $329,021, resulting in an ICER of $146409.07/QALY when compared to the chemotherapy group.

 Table  4 presents the cost-effectiveness analysis results for the pembrolizumab group versus the chemotherapy group, considering the complementary drug policy. In this analysis, the average cost for the chemotherapy group was $59,112 per patient, with a mean QALY of 2.63. The pembrolizumab group, on the other hand, showed a clinical benefit increase of 1.69 QALY but incurred a higher cost of $153,893. This resulted in an ICER of $56127.74/QALY in comparison to the chemotherapy group.

**Table 2:** K-M survival curve fitting results for PFS and OS in the pembrolizumab and chemotherapy groups

Group	Survival Curve	exponential		weibull		Loglogistic		Lognormal	
		AIC	BIC	AIC	BIC	AIC	BIC	AIC	BIC
Pembrolizumab	PFS	793.270	796.274	789.768	795.776	784.079	790.087	780.672	786.680
	OS	493.965	496.969	495.729	501.737	493.763	499.771	491.197	497.204
Chemotherapy	PFS	1518.997	1522.704	1512.191	1519.605	1480.676	1488.090	1477.743	1485.157
	OS	1451.764	1455.471	1433.157	1450.571	1430.543	1437.957	1421.995	1429.409

**Table 3:** Survival curve distribution parameters

Group	Survival Curve	Distribution parameters
Pembrolizumab	PFS	Meanlog = 2.69410;sdlog = 1.02422
	OS	Meanlog = 4.05435;sdlog = 1.48690
Chemotherapy	PFS	Meanlog = 2.212436;sdlog = 0.968017
	OS	Meanlog = 3.30221;sdlog = 1.05719

**Table 4:** Results of cost-utility analysis

Treatment	COST	LYs	QALYs	C/E	ΔCOST	ΔLYs	ΔQALYs	ICER
Free medication is not considered								
Chemotherapy	81,784	4.89	2.63	91886.97	—	—	—	—
Pembrolizumab	329,021	8.87	4.32	187960.62	247,237	3.99	1.69	146409.07
Consider medication giveaways								
Chemotherapy	59,112	4.89	2.63	63789.80	—	—	—	—
Pembrolizumab	153,893	8.87	4.32	84001.05	94,781	3.99	1.69	56127.74

### Sensitivity analysis

**Fig. 3:**
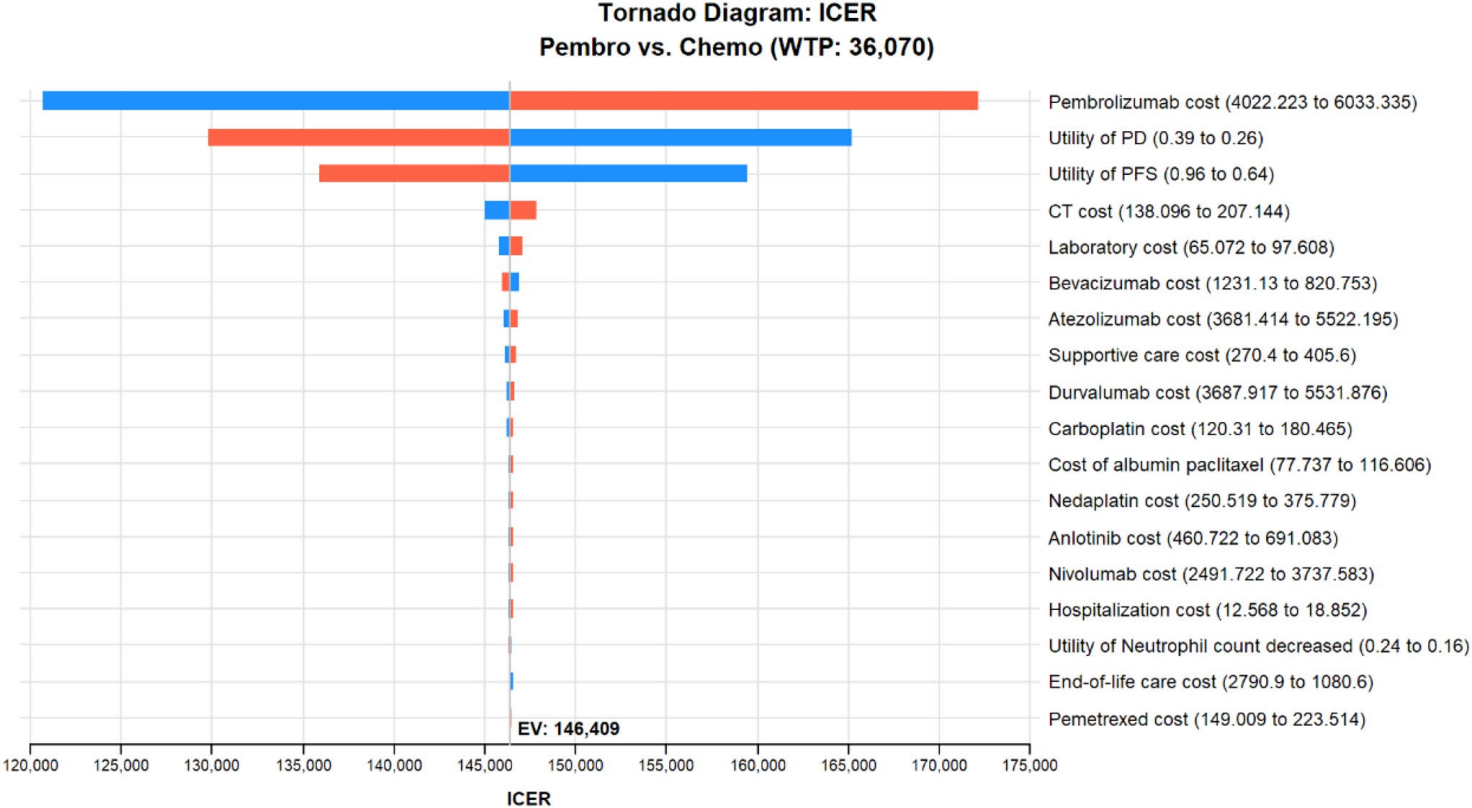
Tornado chart for one-way sensitivity analysis (Free medication is not considered)

**Fig. 4:**
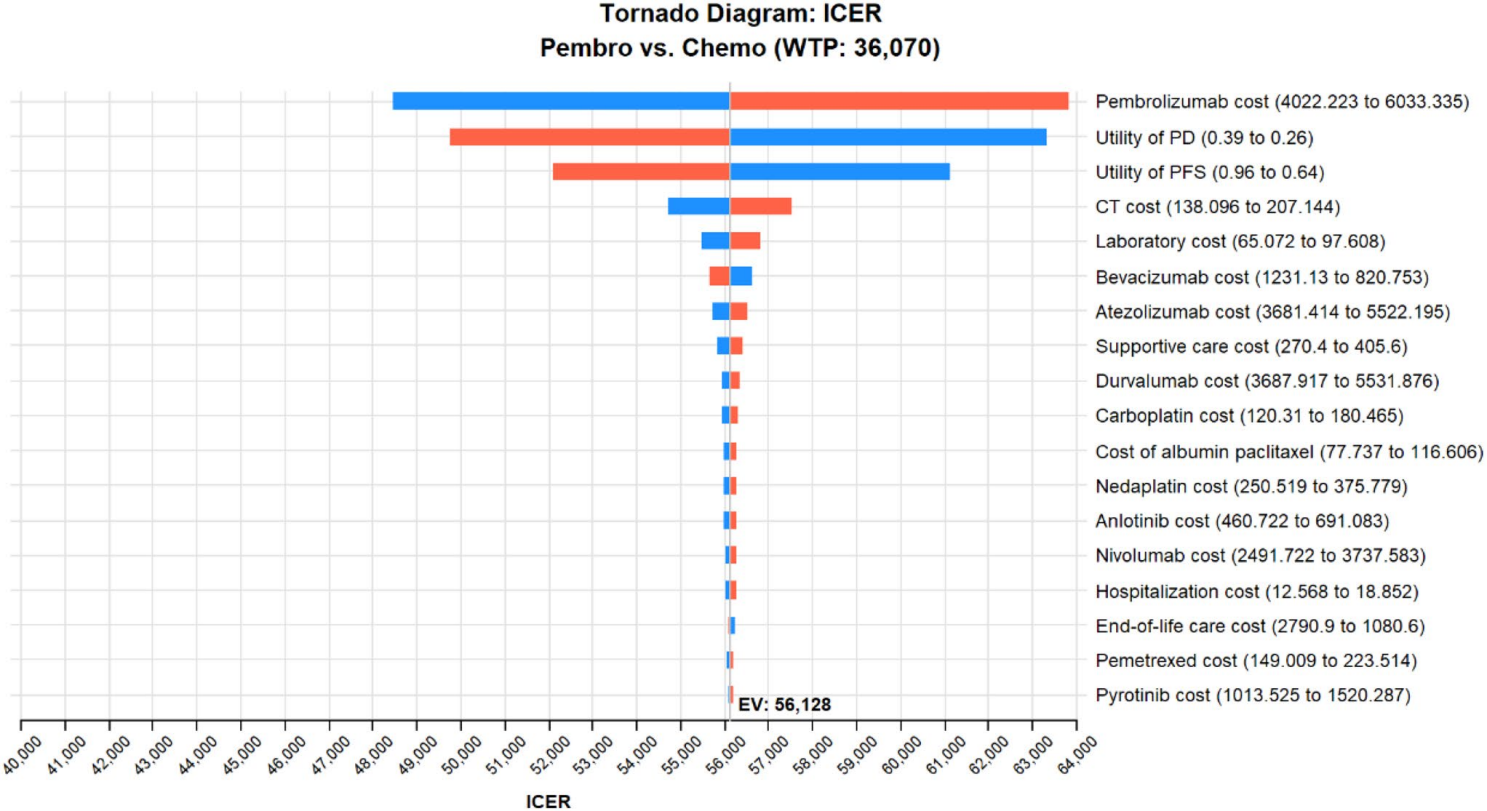
Tornado chart for one-way sensitivity analysis (Consider Medication giveaways)

The study conducted a DSA on several cost parameters including drug costs, utility values for two disease states, associated screening and treatment expenses, and utility values for adverse effects, with variations set at ± 20% of their base values. The WTP threshold was set at three times China’s GDP per capita for the year 2022. The findings indicated that the price of pembrolizumab, the utility values associated with disease progression and non-progression, and the cost of CT scans significantly influenced the outcomes, both with and without the consideration of complementary drugs. These results are depicted in Figs. 3 and 4. It was also noted that a reduction in the price of pembrolizumab would lead to a further decrease in the ICER values.

**Fig. 5:**
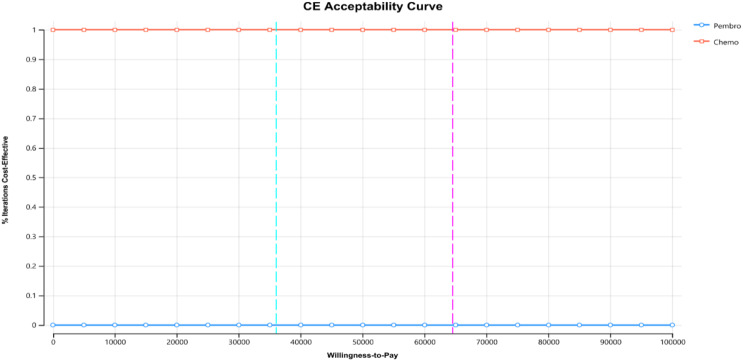
Curves of cost-effectiveness acceptability (Free medication is not considered)

**Fig. 6:**
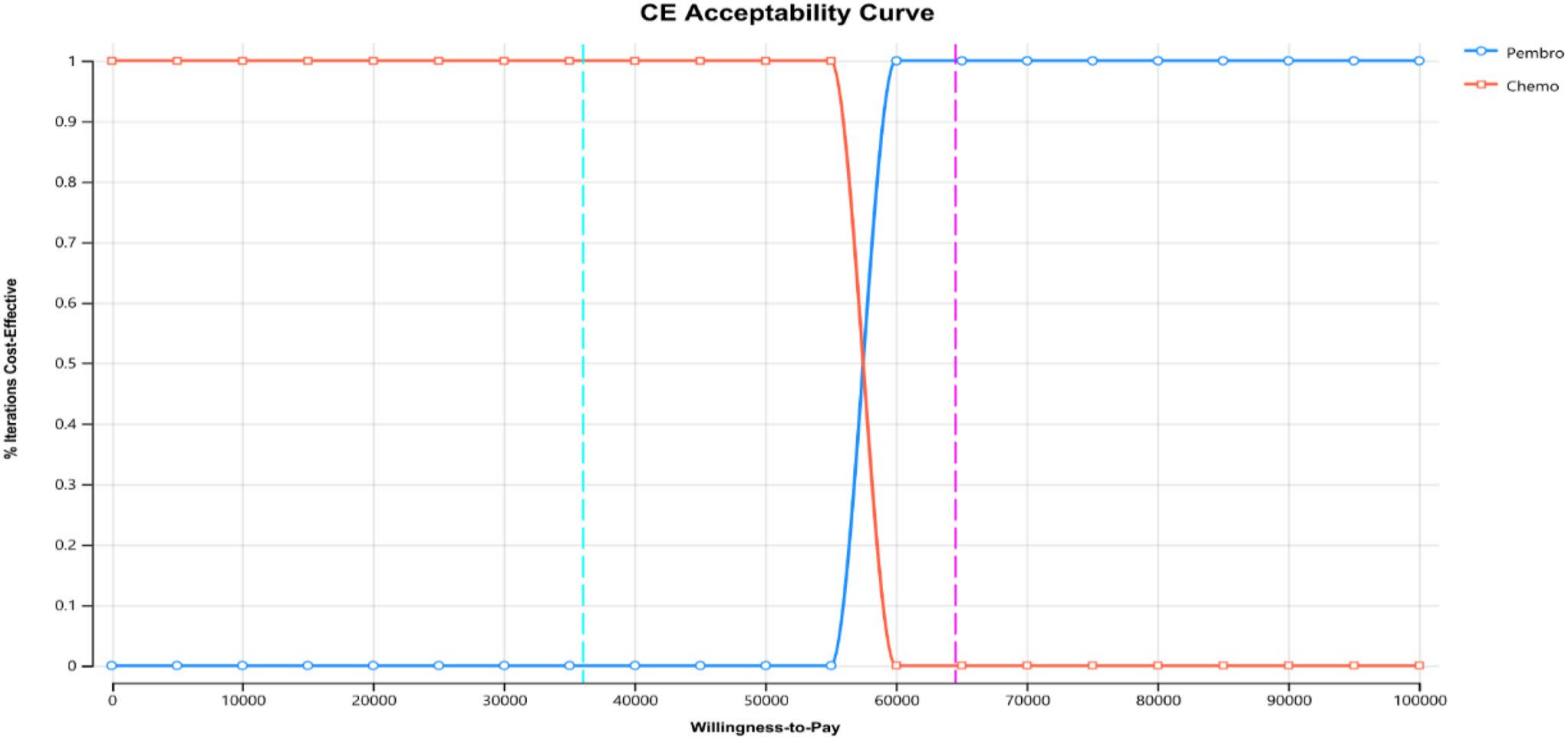
Curves of cost-effectiveness acceptability (Consider Medication giveaways)

This study conducted a PSA through 1000 Monte Carlo simulations, as shown in Figs. 5 and 6.

From the cost-effectiveness acceptability curves, it’s evident that when comparing the pembrolizumab group (without complimentary drugs) and the chemotherapy group, as well as the pembrolizumab group (with complimentary drugs) against the chemotherapy group, the chemotherapy group attains 100% acceptability at a cost threshold of approximately three times China’s GDP per capita ($36070.2), outperforming the pembrolizumab group. Additionally, in the pembrolizumab group with complimentary drugs, its cost-effectiveness acceptability curve relative to the chemotherapy group indicates that at a threshold of three times Guangzhou’s per capita GDP ($64523.8), pembrolizumab achieves 100% acceptability.

## Discussion

 PD-1/PD-L1 antibody inhibitors have become an important antitumor agent after surgery, radiotherapy, and chemotherapy due to their excellent clinical efficacy (Chen 2020; Peng et al. 2021; Planchard et al. 2020; Ruiz-Patiño et al. 2020; Sanaei et al. 2021; Wan et al. 2019). Compared with conventional chemotherapy, pembrolizumab has clinically significant efficacy, but its treatment cost is higher and needs further economic evaluation. In our research, we assessed the cost-effectiveness of pembrolizumab monotherapy and combination chemotherapy treatment compared to chemotherapy treatment, utilizing real-world data. We employed a partitioned survival model for this evaluation. The study period was the entire life cycle of the patients. This study is from the perspective of the health care system and only direct medical costs were calculated for patients’ treatment costs. The study results showed that the cost of treatment required to achieve a clinical benefit of 1 QALY was $187960.62 and $91886.97 for the pembrolizumab and chemotherapy groups respectively, when the drug-grant policy was not considered. In contrast to the chemotherapy group, opting for pembrolizumab monotherapy or a combination of chemotherapy treatments can result in superior clinical benefits. The ICER for these treatments amounts to $146409.07/QALY, which significantly exceeds the threshold set at three times China’s per capita GDP in 2022. Therefore, the investment in treatment costs to attain additional clinical benefits in the pembrolizumab group may not be considered cost-effective. Comparing this study with RCT-based economics studies, this study was consistent with published economics studies concluding that it is not economical in China (Cai et al. 2021; Jiang and Wang 2022; Shi et al. 2021; Zhou et al. 2019 ). For example, the results of a study based on KEYNOTE0-024 showed that pembrolizumab did not have a cost-effectiveness advantage in China, and that the utility value of PFS, the cost of pembrolizumab, and the utility value of PD were the factors that had the greatest impact on ICER (Liao et al. 2019). Another study based on KEYNOTE-189 showed that the PD-L1 testing regimen was not cost-effective in China, and the utility value of PD and the price of pembrolizumab had the greatest impact on ICER values (Wan et al. 2020). None of the studies listed above took into account the complimentary drug policy. In contrast, in our study, we additionally considered the complimentary drug policy. The pembrolizumab group was still not economical under the three times GDP per capita threshold when the drug gift policy was considered. Since the cost data in this study were mainly from Guangzhou, the pembrolizumab group was economical under the threshold of three times the per capita GDP in Guangzhou, thus, it can be seen that the economics of drugs can be improved to some extent under the premise of enjoying the complimentary drug policy in developed areas.

Several economic limitations should be noted in this study. Firstly, This economic evaluation has certain limitations. Firstly, real-world data sourced from hospital HIS medical records systems may not be comprehensive enough and could potentially suffer from incomplete medical records and inconsistent data quality, thereby affecting the accuracy and reliability of the study results. Secondly, the non-randomized allocation of patient populations in real-world studies may lead to selection bias. Thirdly, the sensitivity analysis outcomes indicated that utility values corresponding to disease progression and its absence played crucial roles and could potentially bias the results of the economic assessment conducted in this study. Fourthly, limitations of this study include the use of traditional P-value tests rather than standardized effect sizes (e.g., SMD) for between-group comparisons. Subsequent extension studies will use the SMD method for sensitivity analyses to enhance the comparability and interpretive power of the results. Therefore, there is an urgent need to further refine the measurement of utility values among different treatment modalities in the Chinese population in the future.

## Conclusions

Our findings demonstrated that when considering a WTP threshold set at three times the GDP per capita ($36070.2) in China, the pembrolizumab treatment group did not exhibit a cost-effectiveness advantage compared to the chemotherapy regimen in patients with advanced NSCLC. This economic disadvantage persisted even when considering the benefit of the drug-grant policy. However, the pembrolizumab group was economic when the threshold was set at three times the per capita GDP ($64523.8) in Guangzhou, considering the drug-grant policy. The economics of pembrolizumab can be improved to some extent by considering lowering its price or using it in developed areas in the future.

## Electronic supplementary material

Below is the link to the electronic supplementary material.

Supplementary Material 1

## Data Availability

No datasets were generated or analysed during the current study.
