# Supplementary material for: Cost-effectiveness analysis of pembrolizumab versus chemotherapy in advanced non-small cell lung cancer in China based on real-world studies
Source: J Cancer Res Clin Oncol. 2025 Oct 28;151(12):306. doi: 10.1007/s00432-025-06242-6 (PMC12569207; doi:10.1007/s00432-025-06242-6)
Supplement: Supplementary file 1 — Supplementary Material 1 [file 432_2025_6242_MOESM1_ESM.docx]

Supplementary materials

**TABLE S1** Cost of drug treatment in the pembrolizumab and chemotherapy groups.

| Treatment Cost | Base case ($, per cycle) | Lower limit ($) | Upper limit ($) | Distribution |
| --- | --- | --- | --- | --- |
| Pembrolizumab | 5027.779 | 4022.22 | 6033.335 | Gamma |
| Anlotinib | 575.902 | 460.722 | 691.083 | Gamma |
| Paclitaxel (Albumin Bound) | 97.172 | 460.722 | 691.083 | Gamma |
| Recombinant | 867.094 | 693.675 | 1040.513 | Gamma |
| Gemcitabine | 344.029 | 275.223 | 412.835 | Gamma |
| Bevacizumab | 1025.941 | 820.753 | 1231.130 | Gamma |
| Lobaplatin | 453.547 | 362.837 | 544.256 | Gamma |
| Cisplatin | 47.800 | 38.240 | 57.360 | Gamma |
| Pemetrexed | 186.262 | 149.009 | 223.514 | Gamma |
| Paclitaxel Liposome | 259.105 | 207.284 | 310.927 | Gamma |
| Nedaplatin | 313.149 | 250.519 | 375.779 | Gamma |
| Docetaxel | 28.228 | 22.583 | 33.874 | Gamma |
| Carboplatin | 150.387 | 120.310 | 180.465 | Gamma |
| Etoposide | 34.093 | 27.274 | 40.911 | Gamma |
| Camrelizumab | 361.496 | 289.197 | 433.795 | Gamma |
| Tislelizumab | 386.526 | 309.220 | 463.831 | Gamma |
| Toripalimab | 268.253 | 214.602 | 321.904 | Gamma |
| Nimotuzumab | 402.660 | 322.128 | 483.192 | Gamma |
| Sintilimab | 303.047 | 242.438 | 363.657 | Gamma |
| Tegafur, Gimeracil and Oteracil Potassium | 34.654 | 27.723 | 41.585 | Gamma |
| Pyrotinib Maleate | 1266.906 | 1013.525 | 1520.287 | Gamma |
| Alectinib | 70.978 | 56.782 | 85.173 | Gamma |
| Durvalumab | 4609.897 | 3687.917 | 5531.876 | Gamma |
| Nivolumab | 3114.653 | 2491.722 | 3737.583 | Gamma |
| Atezolizumab | 4601.830 | 3681.464 | 5522.195 | Gamma |
| Cetuximab | 757.618 | 606.095 | 909.142 | Gamma |
| Afatinib | 137.494 | 109.995 | 164.992 | Gamma |
| Crizotinib | 852.363 | 681.890 | 1022.835 | Gamma |
| Almonertinib | 593.973 | 475.178 | 712.767 | Gamma |
| Osimertinib | 487.724 | 390.179 | 585.269 | Gamma |
| Axitinib | 1219.765 | 975.812 | 1463.719 | Gamma |
| Inetetamab | 215.220 | 172.176 | 258.264 | Gamma |
| Vinorelbine Tartate | 591.083 | 472.866 | 709.299 | Gamma |

**TABLE** **S2** Other associated treatment costs for the pembrolizumab and chemotherapy groups.

| Other Treatment Cost | Base case ($, per cycle) | Lower limit ($) | Upper limit ($) | Distribution |
| --- | --- | --- | --- | --- |
| Neutropenia prevention or Treatment | 428.530 | 324.824 | 514.236 | Gamma |
| Thrombocytopenia prevention or treatment | 1473.480 | 1178.784 | 1768.176 | Gamma |
| Anemia prevention or treatment | 493.710 | 394.968 | 592.452 | Gamma |
| TPS/Genetic Screening | 201.950 | 161.560 | 242.340 | Gamma |
| Hospitalization | 15.710 | 12.568 | 18.852 | Gamma |
| Laboratory test | 81.340 | 65.072 | 97.608 | Gamma |
| CT examination | 172.620 | 138.096 | 207.144 | Gamma |
| Supportive care | 338.000 | 270.400 | 405.600 | Gamma |
| End-of-life care | 2325.750 | 1860.600 | 2790.900 | Gamma |

**TABLE** **S3** Utility value parameters for the pembrolizumab and chemotherapy groups.

| Utilities | Value | Lower limit | Upper limit | Distribution |
| --- | --- | --- | --- | --- |
| PD | 0.321 | 0.257 | 0.385 | Beta |
| PFS | 0.804 | 0.643 | 0.965 | Beta |
| Neutropenia | 0.200 | 0.160 | 0.240 | Beta |
| Thrombocytopenia | 0.110 | 0.088 | 0.132 | Beta |
| Anemia | 0.070 | 0.056 | 0.084 | Beta |
